# Childhood overweight and obesity in a region of Italian immigration in Southern Brazil: a cross-sectional study

**DOI:** 10.1186/s13052-015-0126-6

**Published:** 2015-04-02

**Authors:** Renata Geremia, Hosana Maria Speranza Cimadon, William Brasil de Souza, Lucia Campos Pellanda

**Affiliations:** Programa de Pós-Graduação em Cardiologia, Fundação Universitária de Cardiologia, Porto Alegre, Brazil; Centro Universitário Feevale, Novo Hamburgo, Brazil; CNEC, Bento Gonçalves, Brazil; Universidade Federal de Ciências da Saúde de Porto Alegre (UFCSPA), IC/FUC, Avenida Princesa Isabel, 370, Porto Alegre, RS 90620-001 Brazil

**Keywords:** Overweight, Obesity, Children, Adolescents, Prevention

## Abstract

**Background:**

The main modifiable risk factors for obesity are related to lifestyle and significantly influenced by the family, environment and culture. We aimed to estimate the prevalence of overweight/obesity and associated lifestyle factors in children from Bento Gonçalves, a southern Brazil city with strong Italian immigration influence. Italian traditional foods were locally adapted since the immigrants’ arrival in the XIX century, to include more fat and fewer vegetables, and physical activity levels have decreased.

**Methods:**

Cross-sectional study of a population-based cluster sample with students aged 9–18 years. We assessed time spent in sedentary behaviors, hours of physical activity, food frequency and family history. All children underwent physical examination with anthropometric and blood pressure measurements. Overweight and obesity were classified according to WHO percentile curves.

**Results:**

A total of 590 students were evaluated. Mean age was 12.45 ± 1.49 years. The prevalence of overweight and obesity was 16.3% and 8.3%, respectively. Boys were more frequently overweight and obese than girls (16.3% and 12.2% versus 16.2% and 5.5%, respectively). Vegetables and fruits were consumed less than 4 times per week in 49% and 36.8%, while soft drinks, fast food and sweets were consumed more than 4 times a week by 71%, 70.3% and 42.7%, respectively. The habit of omitting breakfast was associated with overweight (p = 0.007). The average screen time was 5.38 ± 2.88 hours/day. Overweight/obesity was present in 12.2% (n = 5), 24.8% (n = 122) and 36.8% (n = 14) children with low birth weight, normal birth weight and high birth weight respectively (*p* = 0.04). The prevalence of high blood pressure was higher in obese (30.6%) and overweight (21.2%) children, comparing to eutrophic children (6.8%; p < 0.001). Excess weight was more frequent among fathers (62.8%) than in mothers (46.3%), but excess weight in mothers was positively associated with excess weight in children (p 0.048).

**Conclusion:**

The city showed high prevalence of overweight and obesity. These findings reinforce the importance of implementing prevention strategies aimed at children and their families, considering that health habits are shared and transmitted along generations.

## Background

Overweight and obesity have increased substantially over the last decades. Worldwide, it has been estimated that 170 million children are overweight [1].

Rates of obesity in youth are expected to continue to grow, leading to important consequences in psychological and physical health [2-17].

Several causes may be related to this increase in childhood obesity. The prevalence of overweight and obesity is associated with birth weight, parents’ BMI, child’s nighttime sleep duration, early solid foods initiation, lifestyle that includes decreased physical activity, increased media use, sedentary behavior in general and unfavorable changes in eating habits [10-17].

The main modifiable risk factors for obesity are related to lifestyle and are significantly influenced by the family environment [18-23]. Therefore, local culture may be an important factor in this scenario. Migration patterns offer the opportunity to study interactions between the original and local cultures. Bento Gonçalves, a city founded by 730 Italian immigrants in 1875, is located in Rio Grande do Sul, the southernmost state of Brazil, and has a population of approximately 111,000 inhabitants. Although its population is mainly Italian in origin, with strong Italian cultural influence, it has adapted to Brazilian culture. Previous studies have shown an increasing prevalence of obesity in other `regions of Brazil [24-27] and in Rio Grande do Sul [28-31], but not under these particular conditions. We have already shown that the most consumed protein in this population was beef, with a low consumption of fish, vegetables, and fruits [32].

Thus, the objective of the present study was to describe lifestyle factors associated with overweight and obesity in children from this particular region of Brazil.

## Population and methods

We conducted a population-based, cross-sectional study with probabilistic cluster sampling, stratified by type of school (public or private) in the urban area of the city of Bento Gonçalves (92.4% of the city dwell in the urban area). The total city population was of 107.278 inhabitants in 2010 (with Self-perceived skin colour as declared to Brazilian Census: White 87.41%; Black 2.05%; Yellow 0.21%; Native Brazilian 96 0.08%; and Mixed 10.962 10.21%) [33]. The human development index (HDI) of Bento Gonçalves is 0,778, the 145^th^ position among Brazilian cities [6].

Schools were drawn from the listing of all city schools, public and private, provided by Education Council.

Inclusion criteria were predefined as follows: children and adolescents who were attending school between 5th and 8th grades of Fundamental School and with less than 18 years old. Children who had genetic, orthopedic or other health conditions that would preclude physical examination were excluded.

Sample size was determined with the help of the EPI INFO (StatCalc) software. Considering that a previous similar study from our group [29] determined a 9.8% prevalence of obesity in Porto Alegre - RS, we estimated that the sample should include 415 students, with a confidence level of 95% and margin of error of 2.7%. This size was increased in 30% for possible losses, totaling 539 students.

The project was approved by the Instituto de Cardiologia Research Ethics board, and was also submitted and approved by the city of Bento Gonçalves department of education. An overview of the program objectives and activities was presented to each of the selected schools, with discussion of the study project with the administration board and faculty. A pilot study was conducted in one school, allowing adjustments in the instrument of data collection. After signature of the informed consent by the parents or guardians and students, they received a questionnaire containing questions about family history, history of pregnancy, birth and breastfeeding, eating habits and lifestyle, and a food frequency questionnaire previously validated in Brazil [34].

After returning the completed questionnaire, the students were evaluated for anthropometric measurements and blood pressure.

Body weight was measured on a 120-kg capacity calibrated portable electronic scale, with the child standing in the center of the scale barefoot and wearing only light clothing, with arms outstretched to the side and not moving. Height was obtained using a tape measure with a precision of 0.1 cm, fixed on smooth walls without footer, and a triangle. The children were placed erect, with parallel feet and heels, and shoulders and buttocks touching the wall.

Systemic blood pressure was measured three times, after a 10 minute rest, with the use of an aneroid manometer, range 0 to 300 mm Hg, as indicated in The fourth report on the diagnosis, evaluation, and treatment of high blood pressure in children and adolescents [35]. The lowest value of the three was used for classification. Elevated blood pressure was defined as systolic or diastolic pressure above the 90th percentile for age and height [35]. However, since all three measures were taken in the same occasion, children with elevated values did not receive a definite diagnosis of hypertension, but were referred to the health unit for further evaluation.

The body mass index (BMI) was calculated as weight in kilograms divided by height in square meters, and expressed as kg/m [18]. For classification of nutritional status, cutoff points for overweight (between percentiles 85 and 97) and obesity (above the 97^th^ percentile) were in accordance to the guidelines of the World Health Organization, 2007 [36,37]. Students below the 85^th^ percentile are indicated as reference category.

Dietary habits were investigated using a food frequency questionnaire. Information was collected regarding the number of daily meals, if breakfast was consumed, and frequency of consumption of specific foods, such as fruits, vegetables, fast food, sweets and beverages. Detailed description of the food frequency questionnaires is available elsewhere [32].

Information regarding time spent in sedentary behavior (television/video game/computer) and weekly hours of physical activity (supervised or unsupervised physical activity) were collected.

Birth weight was collected from the child card that every Brazilian child receives at birth, and considered high when it exceeded the 4.000 g, and low when less than 2,500 g.

Data were analyzed with SPSS (version 20.0) statistical software. Quantitative variables are described as mean and standard deviation or median and interquartile range. Chi-square test was used for qualitative variables, with adjusted residual analysis used as a complement, when necessary. The mean of quantitative variables that satisfied the normality assumption regarding the classification of nutritional status were compared with analysis of variance. When significant differences were observed, the Tukey test for multiple comparisons was used. The correlation between nutritional status and number of meals was analyzed using the Spearman correlation coefficient. Poisson regression analysis was used to evaluate factors independently associated with excess weight. In all comparisons, a 5% significance level (p < 0.05) was considered to be statistically significant.

## Results

A total of 590 students, from 10 schools, were evaluated. Mean age was 12.45 ± 1.49 years, and 58.5% (n = 345) were female. The students attended public (90.2%, n = 532) and private schools (9.8%, n = 58), corresponding to the proportion of public and private schools in the city. Characteristics of the sample and lifestyle behaviors of the students are presented in Table 1.

**Table 1:** **General characteristics and lifestyle variables**

**Variables**	
Students	
Male	245 (41.5)
Female	345 (58.5)
Age	
9 to 13 years	459 (77.8)
14 to 18 years	131 (22.2)
Education level of father	
Illiterate/did not attend school	6 (1.2)
Incomplete or complete elementary school	314 (61)
Incomplete or complete secondary grade school	150 (29.1)
Incomplete or complete college or higher education	45 (8.7)
Education level of mother	
Illiterate/did not attend school	1 (0.2)
Incomplete or complete elementary school	331 (59.2)
Incomplete or complete secondary grade school	162 (29)
Incomplete or complete college or higher education	65 (11.6)
Number of daily meals	4.44 ± 0.98
Breakfast consumption	426 (72.2)
Frequency of consumption	
Vegetables < 4 times per week	292 (49.5)
Fruits < 4 times per week	215 (36.8)
Sugar-sweetened beverages* ≥ 4 times per week	419 (71)
Fast food^†^ ≥ 4 times per week	411 (70.3)
Sweets^‡^ ≥ 4 times per week	252 (42.7)
Physical activity < 3 times per week	306 (52.3)
Hours spent with television/video game/computer per day	5.38 ± 2.88

n (%); Mean ± SD; *Sugar-sweetened beverages: regular soda, juice powder, concentrated juice, natural juice with sugar; ^†^Fast food: empanadas, pizza, hot dogs, french fries, fried pastry, cheeseburgers”; ^‡^Sweets: candy, sandwich cookies, chocolate, crisps.

The prevalence of overweight and obesity was respectively 16.3% (95% CI 13.3 to 19.3) and 8.3% (95% CI 6.1 to 10.5).

The frequency of obesity was higher among boys (12.2%, n = 30) than girls (5.5%, n = 19). Table 2 describes general characteristics and lifestyle variables according to gender. There was a significant difference in physical activity levels: 46.3% of boys and 56.6% of girls reported to practice physical activities less than 3 times a week (0.018). A difference was also observed regarding sweets consumption: 47.8 of girls and 35.5% of boys reported to consume more than 4 times a week (0.004).

**Table 2:** **General characteristics and lifestyle variables according to gender**

**Variables**	**Male (n = 245)**		**Female (n = 345)**		**p**
	**N**	**%**	**n**	**%**	
Age					0.305
9 to 13	185	75.5	274	79.4	
14 to 18	60	24.5	71	20.6	
Birth weight					0.024
<2500 g	16	6.7	25	7.6	
2500 g < and < 4000 g	200	83.3	292	88.2	
>4000 g	24	10.0	14	4.2	
Breakfast consumption	180	73.5	246	71.3	0.628
Frequency of consumption					
Vegetables < 4 times per week	122	49.8	170	49.3	0.967
Fruits < 4 times per week	79	32.5	136	39.8	0.088
Sugar-sweetened beverages* ≥ 4 times per week	168	68.6	251	72.8	0.312
Fast food ≥ 4 times per week	171	70.4	240	70.2	1.000
Sweets ≥ 4 times per week	87	35.5	165	47.8	0.004
Physical activity < 3 times per week	113	46.3	193	56.6	0.018
Hours spent with television/video game/computer per day	5.63*	2.94**	5.21*	2.82**	0.423

*mean **standard deviation.

There was a significant but weak (r_s_ = − 0.108; p = 0.009) inverse correlation between nutritional status and the number of meals per day.

The habit of omitting the breakfast was associated with overweight (p = 0.007) (Table 3).

**Table 3:** **Characteristics of students according to nutritional status**

**Characteristics**	**Nutritional status**						**P**
	Reference*		**Overweight**		**Obesity**		
	**n**	**%**	**n**	**%**	**n**	**%**	
Gender							
Male	175	71.4	40	16.3	30	12.2^†^	0.013
Female	270	78.3^†^	56	16.2	19	5.5	
Age (years)							
9 to 13	340	74.1	80	17.4	39	8.5	0.318
14 to 18	105	80.2	16	12.2	10	7.6	
Breakfast consumption	336	78.9^†^	59	13.8	31	7.3	0.007
Physical activity < 3 times per week	222	72.5	55	18.0	29	9.5	0.246
Elevated blood pressure							
Absent	411	79.2^†^	74	14.3	34	6.6	<0.001
Present	30	46.2	20	30.8^†^	15	23.1^†^	

p = minimal significance level of the chi-square test; *Low and normal weight; ^†^Adjusted residual analysis: p < 0.05.

Lack of physical activity was not associated with overweight and obesity (Table 3). The average number of screen time in hours/day was 5.31 ± 2.80 for eutrophic students, 5.47 ± 3.17 for overweight and 5.90 ± 2 97 for obese students (p = 0.379).

The prevalence of systemic high blood pressure was higher in obese (30.6%) and overweight (21.2%) children, comparing to eutrophic children (6.8%; *p* < 0.001) (Table 3).

Birth weight was directly proportional to current weight, as shown in Table 4. Overweight/obesity was present in 12.2% (n = 5), 24.8% (n = 122) and 36.8% (n = 14) children with low birth weight, normal birth weight and high birth weight respectively (*p* for linear association = 0.04) The prevalence ratio of excess weight in the high birth weight group, when comparing to the normal weight group was of 1.48 (0.95-2.31 p = 0.08). The prevalence ratio of excess weight in the high birth weight group, when comparing to the low weight group was of 3.02 (1.20-7.59 p = 0.012).

**Table 4:** **Association between present status of weight x birth weight**

	**Overweight and obesity childhood**				**p for linear association**
	**Absent**		**Present**		
	**N**	**%**	**N**	**%**	
Birth weight					0,040
<2500 g	36	87,8	5	12,2	
2500 g < and < 4000 g	370	75,2	122	24,8	
>4000 g	24	63,2	14	36,8	

p = minimal significance level of the chi-square test.

Overweight and obesity was more frequent among fathers (62.8%, n = 294) than in mothers (46.3%, n = 273). As shown in Table 5, overweight and obesity in mothers, but not fathers, was associated with excess weight in children (*p* 0.0048). In multivariate analysis, age, male gender, breakfast consumption and the mother’s nutritional status remained independently associated with children’s excess weight (Table 6).

**Table 5:** **Association between overweight and obesity of students and overweight and obesity of parents**

	**Overweight and obesity of students**		**P**
	**N**	**%**	
Overweight and obesity of fathers			
Absent	38	21.8	0.135
Present	84	28.6	
Overweight and obesity of mothers			
Absent	67	21.2	0.048
Present	78	28.6	

p = level of significance of the chi-square test.

**Table 6:** **Summary of poisson regression analysis for association between lifestyle variables and excess weight**

**Variable**	**B**	**Std. error**	**OR**	**95% Wald confidence interval for OR**		**p**
				**Lower**	**Upper**	
Age (years)	-.133	.0615	.875	.776	.987	.030
Breakfast consumption	-.403	.1495	.669	.499	.896	0.007
Gender: Male	.293	.1450	1.341	1.009	1.782	0.043
Physical activity < 3 times per week	.219	.1490	1.245	.930	1.667	0.141
Mother - overweight and obesity	.022	.0047	1.022	1.012	1.031	<0.001
Hours spent with television/video game/computer per day	.019	.0258	1.019	.969	1.072	0.471

## Discussion

This population-based cross-sectional study showed high prevalence of overweight and obesity in schoolchildren of a city in southern Brazil with strong Italian influence. Similar results have been previously reported for other southern Brazilian cities [28,29], evidencing that, despite the original cultural habits, adaptation to the new region’s local habits could have a strong influence. Italian traditional foods [38] were locally adapted since the immigrants’ arrival in the XIX century, to include more fat and fewer vegetables, and physical activity levels are in decline among this population.

A study [39] made with young Italian and Spanish adults at the university have shown that young generations seem to give up the traditional Mediterranean dietary pattern, adopting new dietary trends, demonstrating that this fact occurs with descendants both in their own countries as well as in new countries. Since Bento Gonçalves is a city with an agriculture-based economy, along with its Italian influence, it was expected that health habits would be more adequate when compared to others regions of Brazil, showing a “Mediterranean pattern”. However, we observed characteristics of an urban center – sedentary lifestyle, low frequency of consumption of vegetables and high levels of screen time. For example, dietary habits observed were similar to those described in a study conducted in Sao Paulo, a densely populated urban center with approximately 10.7 million people [40].

In the present study, breakfast consumption was associated with a lower prevalence of excess weight, in concordance with the literature [23,26-29].

A sedentary lifestyle may also contribute to overweight and obesity [10-17,41,42]. The habit of watching television for prolonged periods, in addition to restricting physical activity, also leads to increased stimulation for poor dietary habits. In the present study, we observed that 52.3% of students engaged in physical activity less than three times per week. Average time spent on television/video game/computer activities was 5.38 hours per day, much more than that recommended [43], but no association with nutritional status was observed. This lack of association may be due to the huge numbers of children watching television for prolonged periods, with consequent limitation of a comparison group, since Tosseli et al. [12] demonstrated significant correlations of lifestyle and physical activity with children’s weight status.

The frequency of obesity was higher among boys than girls in accordance with other studies [17,44,45] around the world. Comparative studies report higher rates of obesity and overweight in boys and significantly lower levels in girls, mainly in urban areas [17,46,47]. This may be related to concerns about the body image affecting girls in an earlier age [48]. Despite boys being generally more active, they also report more hours of television viewing, and consume less vegetables and more sweet drinks compared to girls with the same age [49]. In our study, boys were more physically active, but also consumed less sweets, and there was no difference regarding hours of TV watching and games. One interesting study in China showed that parents tend to be more permissive with boys than girls, allowing them more access to unhealthy foods and television [50]. This may be the case in the Brazilian setting as well. However, some researchers [51-53] have drawn attention as the isolated use of the BMI for categorization of nutritional status, since the gender difference leads to different body compositions due to hormone levels, muscle mass and fat content for example. So, it is important to consider gender to address the relationship between BMI and nutritional status.

Another aspect that is important in the Brazilian context is the intense miscegenation, a process that did not occur in other American countries with the same intensity. This may dilute differences in health variables related to ethnicity and must be considered when interpreting the results [7-9]. Thus, although one may discuss cultural influences, these usually do not correlate perfectly with genetic ancestry. This makes it difficult to analyse separately ethnic groups in the present study. We may argue that race is a social construct, with limited biological meaning. Human race is viewed as a product of our history and culture and not as a marker of our genes. In this sense, the present study it is more close to an ecological analysis of cultural influences, but with no individual data regarding ethnicity.

Cardiovascular diseases, including dyslipidemia, hypertension, left ventricular hypertrophy and atherosclerosis, are among the main comorbidities associated with obesity [2-5,19,54-57]. Elevated systemic blood pressure was observed in 11.1% of the sample, and an association between the prevalence of overweight and obesity with the presence of elevated systemic blood pressure was observed, as in other studies [2-5,27,29,31,42]. The increase of excess weight in children and adolescents may cause a rise in hypertension and other risk factors that are associated with overweight and obesity [5].

In the present study, birth weight was directly associated with current weight as seen in the study conducted by Oldroyd et al. [11]. Higher birth weight is described to be associated with childhood overweight/obesity [11,58-64]. However, it is important to consider that children with low birth weight may also have a risk for future adult obesity, although the association of low birth weight with obesity and chronic diseases in adult life, through fetal programming, may take a longer period to become evident. Thus, is important to monitor closely both sides of this spectrum, low and high birth weight, specially considering that there are many risk interactions during childhood, such as the dietary habits described in the present study, that may accumulate during the life course. Childhood is a critical window for primordial intervention, both due to developmental plasticity and potential of establishing lifelong healthy habits. In addition, Oldroyd [11] showed that high birth weight was associated with a higher risk of overweight/obesity among both gender, however low birth weight was associated with a lower risk of child overweight/obesity in girls but not in boys taking another point to be noted.

Our evaluation of the nutritional status of parents showed that overweight and obesity in students were associated with overweight and obese mothers. Similarly, Kleiser et al. [23] reported a strong association between parental overweight and obesity in school children. These findings reinforce the importance of implementing prevention strategies aimed at students and their families, considering that lifestyle is shared between parents and children [65] and that the mothers’ education was found to be a risk factor for obesity [66]. This may be due to the similarity between the preferences for certain foods and lifestyle habits.

Some limitations inherent to a population-based cross-sectional study merit discussion. Cross-sectional studies do not allow the observation of changes over time, so that a causal relationship may not be inferred. Thus, the fact that poor dietary habits were more common in students classified as reference than in those with obesity may be due to reverse causality bias, and must be interpreted with caution. It is possible that obese students are already changing dietary habits, which is not possible to analyze with a cross-sectional design. There is also a possibility that common spread knowledge about obesity related foods may have induced these individuals’ answers. Despite these limitations, the cross-sectional design is the most commonly used designs in population studies, since it does not involve follow-up time, has low cost and provides important epidemiological information for planning public health measures, since the important increase of cardiovascular diseases that will arise once the present young generations will become adults is of great concern [5].

In conclusion, factors such as number of omission of breakfast, overweight and obesity in the mother, age and male gender were associated with excess weight. Obese children also showed higher blood pressure levels. These findings reinforce the importance of implementing prevention strategies aimed at children and their families [67,68], considering that health habits established during childhood have both short term effects during childhood [4], as well as long term effects during adult life [36,54] and may be shared and transmitted along generations [2,3,39,66,69,70].

## Abbreviations

WHO: World Health Organization
HDI: Human development index
BMI: Body mass index
CI: Confidence interval
SD: Standard deviation
